# Giant Cell Tumor of Lower End of Tibia

**DOI:** 10.1155/2013/429615

**Published:** 2013-06-17

**Authors:** Monish Bami, Ashok R. Nayak, Shreepad Kulkarni, Avinash Kulkarni, Rupali Gupta

**Affiliations:** Department of Orthopedics, Shri B M Patil Medical College, Bijapur 586103, India

## Abstract

*Introduction*. Giant cell tumor of bones is an unusual neoplasm that accounts for 4% of all primary tumors of bone, and it represents about 10% of malignant primary bone tumors with its different grades from borderline to high grade malignancy. *Case Report*. A 35-year-old patient presented with complains of pain and swelling in left ankle since 1 year following a twisting injury to his left ankle. On examination, swelling was present over the distal and anterior part of leg and movements of ankle joint were normal. All routine blood investigations were normal. X-ray and CT ankle showed morphology of subarticular well-defined expansile lytic lesion in lower end of left tibia suggestive of giant cell tumor. Histopathology of the tissue shows multinucleated giant cells with uniform vesicular nucleus and mononuclear cells which are spindle shaped with uniform vesicular nucleus suggestive of GCT. The patient was treated by excision, curettage, and bone cement to fill the defect. *Conclusion*. The patient at 12-month followup is doing well and walking without any pain comfortably and with full range of motion at ankle joint with articular congruity maintained and no signs of recurrences.

## 1. Introduction 

Giant cell tumor of bones is an unusual neoplasm that accounts for 4% of all primary tumors of bone, and it represent about (10%) of malignant primary bone tumors with its different grades from borderline to high grade malignancy. Usually, the age of patients ranges from 20 to 55 years, and the peak age incidence is in the third decade of life, with slight female predominance (1.2 : 1) [1]. It is a locally aggressive tumor which involves the ends of long bones in skeletally mature individuals. The common clinical symptoms are pain related to affected bone, swelling, and decreased range of movement in adjacent joint [2]. The diagnosis of giant cell tumor of bones depends mainly on clinical and radiological examination (plain X-ray and CT scan) on the site of the lesion [2]. The treatment of GCT is directed towards local control without sacrificing joint function. This can be achieved by intralesional curettage with autograft reconstruction by packing the cavity of the excised tumor with morselised iliac corticocancellous bone or using bone cement as packaging material for the defect [3].

## 2. Case Report

A 35 yr old male patient presented with complaints of pain and swelling on the distal aspect of the left leg. The patient suffered a twisting injury to the leg one year ago and had mild pain and swelling for which he took treatment from an osteopath. The pain reduced but the swelling persisted. One month ago, the swelling increased in size and was associated with severe pain which was exaggerated on walking. There was no history of fever, night cries, loss of weight, and loss of appetite present. History of massage therapy was present which was done 4-5 times.

On examination, swelling was present over the distal aspect of the leg about 6 × 4 cm with well-defined margins, smooth surface, and bony deep. The skin over the swelling was normal. Tenderness was present over distal tibia. Ankle movements were normal.

**Figure 1:**
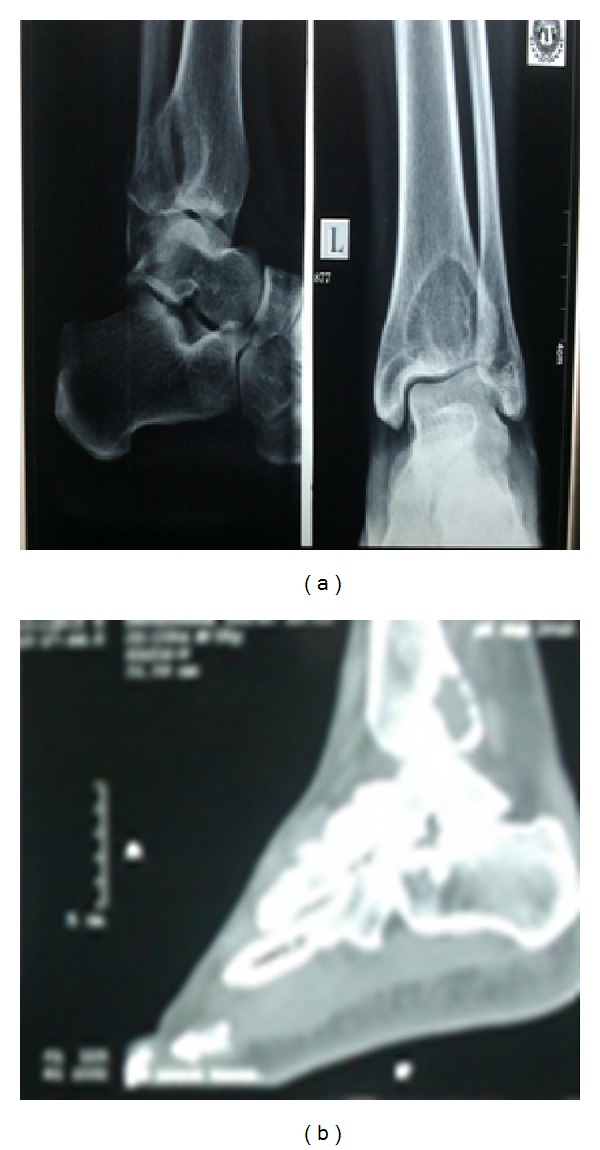
Preop X-ray and CT scan showing osteolytic lesion of distal tibia.

X-ray showed well-defined expansile lytic lesion at distal end of left tibia suggestive of giant cell tumor of distal end tibia. CT (Figure 1) ankle shows morphology of subarticular expansile lytic lesion in lower end of left tibia suggestive of giant cell tumor. 

**Figure 2:**
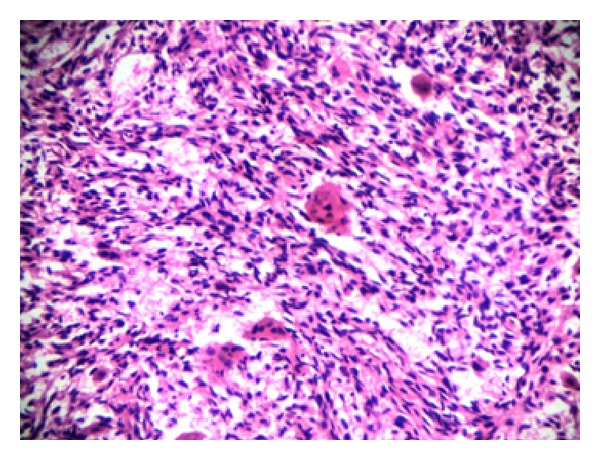
Histopathology of the tumor confirming GCT.

**Figure 3:**
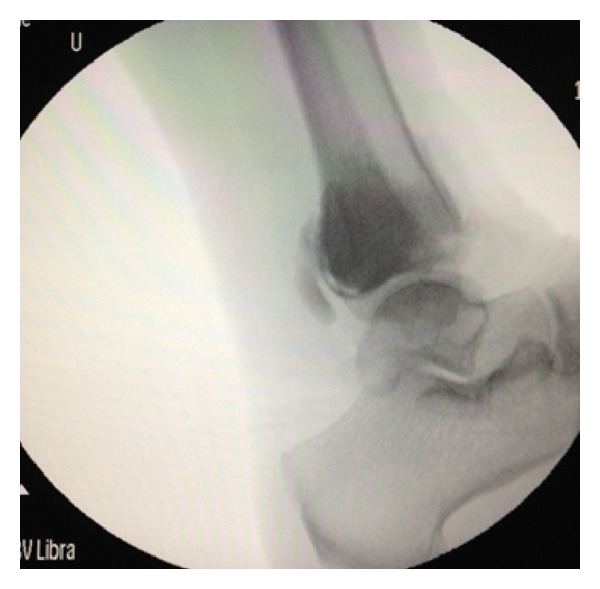
Postoperative radiograph.

The condition and routine investigations done in preparation for operative procedure were explained to the patient. Patient was treated with intralesional excision and curettage and the cavity was filled with bone cement. Specimen was sent for histopathological (Figure 2) examination which confirmed giant cell tumor. The patient was immobilised in an above knee cast for one month and converted to a PTB cast later. Sequential X-rays (Figure 3) were taken to confirm the union and cast removal was done at the end of three months postoperatively. The patient was started on regular physical therapy and was allowed weight bearing when the patient was comfortable. Patient was followed up for one year at regular intervals and the patient was ambulatory without any evidence of recurrence at the end of one year.

## 3. Discussion

Giant tumors are locally aggressive and some may be malignant. The benign form of GCT has the intriguing feature of being able, in rare instances, to metastasize despite otherwise benign characteristics. The malignant variety of GCT has been defined as a sarcomatous growth that is either primarily juxtaposed to a typical benign focus or occurs after a prolonged interval at the site of a previously treated and documented focus [4, 5].

Curettage and wide resection have been the accepted methods of treatment for GCT of bone [6]. Adjuvants such as phenol, hydrogen peroxide used in a percentage varying from 5% to 80% after completion of curettage, may be of additional benefit in helping to decrease recurrence rates after curettage [7, 8]. Some recent studies, though, have questioned the role of adjuvants and filling agents in reducing the recurrence rate of giant cell tumors. Adequate removal of the tumor seems to be a more important predictive factor for the outcome of surgery than the use of adjuvants. The study by Trieb et al. [9] demonstrated that local recurrence rate of giant cell tumors located in long bones treated with or without phenol is similar.

Bini et al. [10] published a paper in which they treated giant cell tumor with curettage and cementation. It is postulated that the exothermic reaction of poly methylmethacrylate generates local hyperthermia which induces necrosis of any remaining neoplastic tissue; yet it does not extend to the normal tissues to result in local complications. The possibility that the polymerization of poly methylmethacrylate may produce a local chemical cytotoxic effect cannot be excluded [11]. It is shown that the addition of cement can reduce the rate of recurrence but is associated with higher risk of subsequent need for joint replacement [12]. Cytotoxic agents like methotrexate and adriamycin have been incorporated in bone cement and other drug delivery systems in an attempt to reduce recurrence [12].

We treated the patient with curettage and use of bone cement to pack the cavity which showed good postoperative results without any recurrences and functional problems. We conclude that it is a good treatment option for GCT of lower tibia.
